# Variations in nutrient and trace element composition of rice in an organic rice-frog coculture system

**DOI:** 10.1038/s41598-017-15658-1

**Published:** 2017-11-16

**Authors:** Zhimin Sha, Qingnan Chu, Zheng Zhao, Yubo Yue, Linfang Lu, Jing Yuan, Linkui Cao

**Affiliations:** 1 School of Agriculture and Biology, Shanghai Jiao Tong University, Shanghai, 200240 China; 2 Institue of Agricultural Resources and Environment, Jiangsu Academy of Agricultural Science, Nanjing, 210014 China; 3 Eco-environmental Protection Institute of Shanghai Academy of Agriculture Science, Shanghai, 201403 China

## Abstract

Introducing frogs into paddy fields can control pests and diseases, and organic farming can improve soil fertility and rice growth. The aim of this 2-year field study was compare the yield and elemental composition of rice between an organic farming system including frogs (ORF) and a conventional rice culture system (CR). The grain yields were almost the same in the ORF system and the CR system. The ORF significantly increased the contents of phosphorus (P), ion (Fe), zinc (Zn), molybdenum (Mo) and selenium (Se) in rice grain at one or both years. However, the ORF system decreased the calcium (Ca) content in grice grains, and increased the concentration of cadmium, which is potentially toxic. A principal components analysis showed the main impacts of ORF agro-ecosystem on the rice grain ionome was to increase the concentration of P and trace metal(loid)s. The results showed that the ORF system is an ecologically, friendly strategy to avoid excessive use of chemical fertilizers, herbicides and pesticides without decreasing yields, and to improve the nutritional status of rice by increasing the micronutrient contents. The potential risks of increasing Cd contents in rice grain should be addressed if this cultivation pattern is used in the long term.

## Introduction

Rice is one of the most important crops to feed the population in China, and its production has dramatically increased over the last 50 years^1^. Improving the yield and quality of rice with minimal chemical fertilizer and pesticide inputs is important for the long-term sustainability of rice production systems over time^1,2^. However, low fertilizer-use efficiency^3–5^, the greenhouse effect^6,7^, water pollution^8,9^, and soil acidification^10^ are problems that caused by intensive paddy field in China. The introduction of ecologically, friendly animals into paddy fields creates an integrated agro-ecological system. Many studies have described rice–fish and rice–duck coculture agro-ecosystems and show how these practices reduced the rate of chemical fertilizer and pesticides^11–14^. However, few studies have focous on the rice–frog coculture system, although this system can effectively pests population of and reduce pollutions in soil and rice grain^15–19^. The rice–frog coculture agro-ecological system, combined with organic farming techniques such as the application of organic fertilizers, represents an ecosystem with effective pest control, less use of chemical fertilizers, and conservation of local soil and water resources^15,20^.

To date, few studies have focused on the cycling of mineral elements, including nutrients and trace elements, in organic farming systems. Application of organic matter can increase the soil fertility, improve the nutrient retention capacity of soil and decrease nutrient losses^21–23^. However, the organic matter application may introduce unwanted metals and metalloids, such as Cd and arsenic (As), because these elements are generally present at higher concentrations in compost than in soil. Such organic compounds can altered soil properties (e.g. pH, redox potential, and organic matter content) to improve the accumulation in plant as well. Previous studies have reported contrasting results with respect to the effects of organic amendments on the uptake of trace elements. Some have reported that addition of organic matter increase the uptake of potentially toxic metals (strontium, Sr; barium, Ba; chromium, Cr; and Cd) by crops^24–27^, whereas others found no significant differences or even a decline in trace metals uptake upon application of organic amendments compared with mineral fertilizer or unfertilized controls^28,29^. Therefore, not only the nutrients but also the nonessential elements need to be evaluated in the soil and crops in organic farming systems. Introducing frogs into the paddy fields has been shown to decrease pest populations and increase the microbial biomass in the soil^15^. However, little is known about the effects of the rice–frog coculture agro-ecological system on the nutrient and trace element contents in rice grains, and it is unknown how this agro-ecosystem affects the grain ionome

Accordingly, the aim of this 2-year field study was to compare the effect of long-term (8 years) conventional rice cultivation (CR) and a rice–frog coculture agro-ecological system (ORF) on (i) the rice grain yield and harvest index, (ii) nutrient and nonessential trace element availability in the soil, and (iii) the protein content and mineral elemental composition of rice shoots and grains. This is the first study on ionome variations in plants and soil in the ORF system, compared with the CR system.

## Materials and Methods

### Experimental site

This 2-year study was conducted in 2013 and 2014 in fields at the Qingpu Modern Agricultural Park, Shanghai, China, which surrounds the Taihu River region in the upper part of the Huangpu River (31°1′N, 121°1′E). During the two rice seasons in 2013 and 2014, the local average temperature was 25.6 °C and 23.8 °C,respectively, and total precipitation was 1178.2 mm and 982.5 mm, respectively. The local soil is classified as Pup-Orthic Entisol according to Chinese Soil Taxonomy, and is derived from lacustrine deposits. There is a high groundwater level in this region. The physical and chemical properties of soils under different cultivation patterns are shown in Table 1. Rice–wheat rotation is the typical cropping system in this area. Most rice plants were transplanted in early June and harvested in late October.

**Table 1:** Physical and chemical properties of soils under different cultivation patterns.

Cultivation patterns	pH	OM	EC	Total N	Available P_2_O_5_	Available K	Organic matter	Water storage	Bulk density	Porosity
	(H_2_O)	(g kg^−1^)	(m S cm^−1^)	(g kg^−1^)	(mg kg^−1^)	(mg kg^−1^)	(g kg^−1^)	(mm)	(g cm^−3^)	(%)
CR	7.3	18.8	0.14	0.91	13.4	101	18.79	23.12	1.36	55.62
ORF	7.5	25.1	0.19	1.21	16.8	127.2	25.09	25.91	1.18	48.62

### Field investigation

The hybrid rice (*Oryza sativa* L.) HuaYou 14, a dominant rice variety in Shanghai, was transplanted into the feild at four weeks after germination, (density of 25 plants.m^−2^), on June 19^th^, 2013. Plant were harvested on September 27^th^, 2013. Rice seedlings of the same cultivar were transplanted into the field at the same density on June 5^th^, 2014, and were harvested on September 13th, 2014.

Two culture systems were established: CR, consisting of a normal rice–wheat rotation system with chemical fertilizer, pesticide, and herbicide application; and ORF, consisting of a rice–frog coculture with organic fertilizer application. No herbicide was applied in the ORF field; instead, the weeds were removed by hand. Chinese milk vetch (*Astragalus sinicus* L.), was seeded in the fields after rice harvest and ploughed into soil the next May as the basal fertilizers. The fields maintained a flooding layer 3–5 cm deep during the rice growing season, and were allowed to dry at the yellow ripening stage. The same rotation (rice–frog coculture and organic amendment) has been applied in the system since 2005. For each system, four plots were established in each year of the study. The area of each field plot was 1600 m^2^, and three sub–plots with area of 100 m^2^ were used for sampling. Table 2 showed the details of two systems, and Table 3 summarizes the types of fertilizers, herbicides and pesticides used.

**Table 2:** Nitrogen fertilizer types and fertilization rates of three cultivation patterns.

Cultivation patterns	Base fertilizer					First dressing			Second dressing		
	Chinese milk vetch	Cloza cake	Mixed seed cake	Biological compound fertilizer	Bulk blending fertilizer	Biological compound fertilizer	Bulk blending fertilizer	Urea	Biological compound fertilizer	Bulk blending fertilizer	Urea
CR	—	—	—	—	75.0	—	75.0	—	—	—	150
ORF	67.5	94.5	36.0	51.8	—	36.5	—	—	29.0	—	—

**Table 3:** Fertilization rates of N, P, K, types of herbicide and pesticide, and the number of introducing frogs in three cultivation patterns.

Cultivation patterns	Herbicides	Pesticides	Frog ×10^4^
CR	Pretilachlor + Paraquat	Abamectin + Triazophos	—
ORF	—	—	2.5

Three or four feeding platforms were placed on each side of the field plot as the resting and feeding places for frogs. Tiger frogs (*Rana tigrina rugulosa*), which are highly adaptable to the environment, was introduced by Zizaiyuan agricultural Development Co., Shanghai. At 10 to 15 days after rice transplanting, frogs large enough (15–20 g) to prey on pests were introduced into the rice fields on sunny mornings (25000 frogs/ha). The animal used in this study were approved by the School of Agriculture and Biology of Shanghai Jiao Tong University, and all animals used in this study were performed in accordance with relevant guidelines and regulations of the Guide for the Care and Use of Laboratory Animals of the Ministry of Health,China.

### Sampling and sample analysis

Uniform plants from each sub plot were sampled for ionome analysis and determination of straw and grain yield. Rice seeds and straws were dried to a constant weight at 70 ^◦^ C after harvest and ground for digestion and analysis according to the method of Chen^30^. Briefly, each 50–mg plant samples was digested in 2 mL of 61% HNO_3_ (EL grade; Kanto Chemical, Tokyo) in a tube for 3 h at 110 °C using a DigiPREP system (SCP Science, Quebec, Canada). Then 0.5 mL of hydrogen peroxide (semiconductor grade; Santoku Chemicals, Tokyo) was added twice at 30 min intervals as an oxidant. The mixture was heated to 110 °C until the solution became clear. After cooling the sample to room temperature, the tubes were filled to 15 mL with 2% HNO_3_ and the concentrations of mineral elements in the digests were analyzed using an inductively coupled plasma mass spectrometer (ICP-MS) (Elan, DRC-e; Perkin-Elmer, Waltham, MA, USA). To estimate crude protein (N × 6.25), total nitrogen (N) was determined by digesting the samples with (H_2_SO_4_ [98%]–H_2_O_2_) and N concentrations were determined using the micro–Kjeldahl method^31^.

At the beginning of the experiment, soil samples were taken from field (0–20 cm) for the basic soil characterization according to the method of Zhao^8^. The results are shown in Table 1. After harvest, soil samples (0–20 cm) were taken from the field to determine the available mineral contents in the soil according to the method of Sha^26^. The available mineral contents were determined by extracting 2 g of air–dried soil with 40 mL 1 M ammonium acetate. The mixture was shaken for 1 h and then filtered through filter paper (No. 5 C). Subsequently, 2 mL of 61% HNO_3_ was added to 5 mL of the filtered extract and concentrated using a DigiPREP system until the solution had evaporated almost completely, using the same procedure as that used to analyze the plant samples. Finally, the tube was filled to 10 mL with 2% HNO_3_ before ICP-MS analysis. In figures and tables, the P, potassium (K), calcium (Ca), and magnesium (Mg) concentrations are expressed in g kg^−1^, and manganese (Mn), Fe, copper (Cu), Zn, boron (B), Mo, nickel (Ni), sodium (Na), Se, aluminum (Al), Cd, Cr, Sr, Ba, and As concentrations are expressed in mg kg^−1^.

### Statistical analyses

Data were tested using analysis of variance (ANOVA) and Tukey’s tests using Stastical Package for the Social Science (SPSS) 22.0 software (SPSS Inc. Chicago, IL, USA). Differences were considered significant at *p < *0.05. A principle component analysis (PCA) was used to profile the protein and mineral element concentrations in the rice grain. The rotation method was varimax with Kaiser normalization.

## Result

### Straw and grain yield and protein content

**Figure 1:**
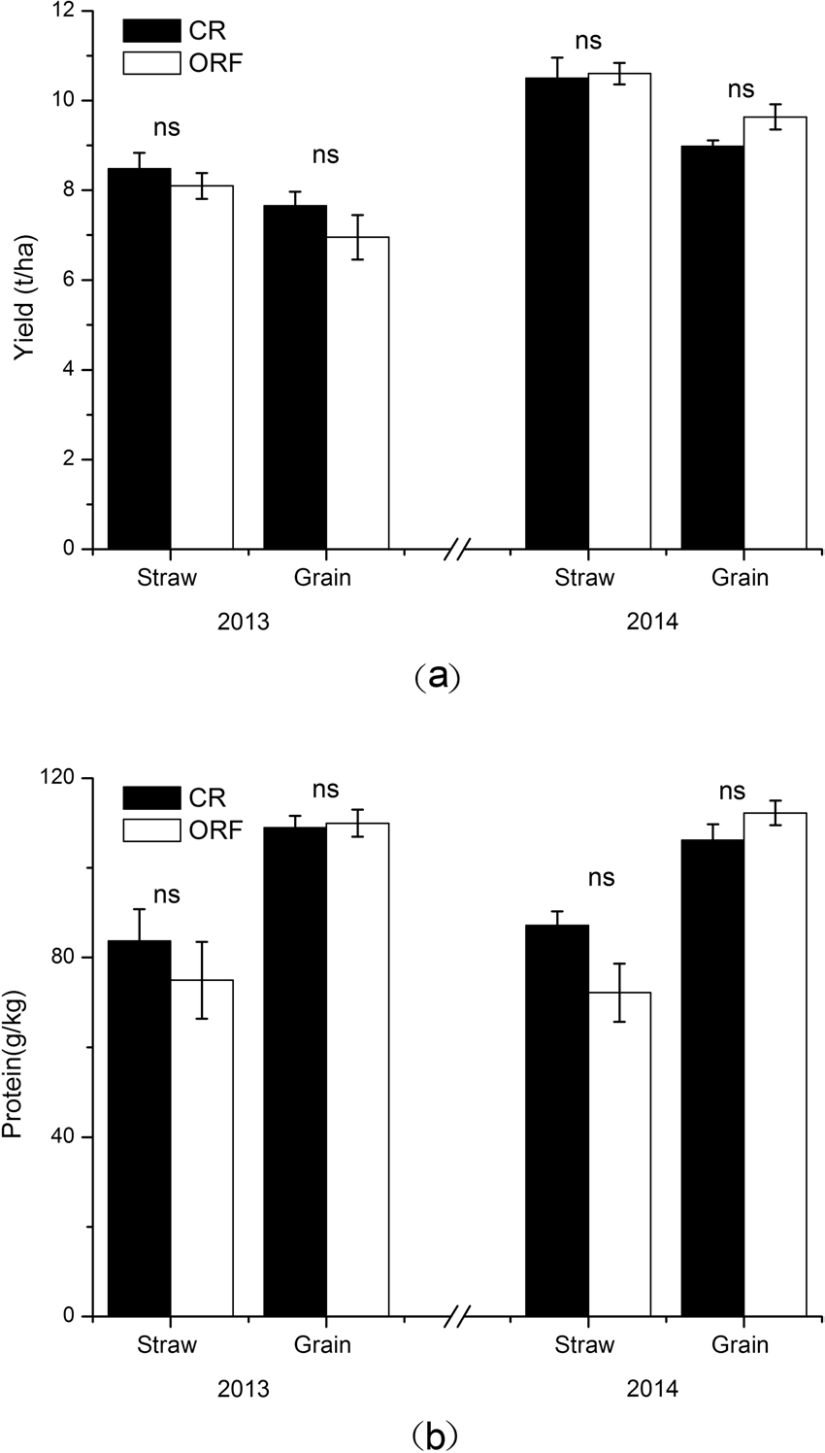
The yield (**a**) and protein content (**b**) in rice straw and grain affected by the conventional rice planting system (CR) and organic rice-frog coculture system (ORF). Values are means ± SE, and “ns” on the bar indicate no significant difference.

The yield and protein content of rice straws and grains did not differ significantly between the CR and ORF systems in either year (Fig. 1). The rice straw yield was higher at 2014 than in 2013. The grain yield was 0.18-fold and 0.39-fold higher in 2013 than in 2014 in the CR and ORF system, respectively.

### Macronutrients concentrations in soil and plant

Table 4 shows the macronutrient content in the soil (available concentrations), straw, and grain in the CR and ORF systems. The ORF system did not significantly affect the K and Mg concentrations in soil or plants The P concentration in soil and grain was significantly higher in the ORF system than in the CR system in both years. In contrast, the Ca concentration in soil and grain was significantly lower in the ORF system than in the CR system in both years. The P concentration in grain was 0.48- and 0.58-fold higher in the ORF system than in the CR system in 2013 and 2014, respectively, but the Ca concentration in grain was 0.58- and 0.63-fold lower in the ORF system than in the CR system in 2013 and 2014, respectively.

**Table 4:** The effects of CR and ORF cultivation patterns on the macronutrient availabilities in the soil (extracted with 1 M ammonium acetate), concentrations in the straw and grain at 2013 and 2014. In the table, calculated by ANOVA with Tukey test (p < 0.05), differences between treatments were significantly representing by bold-filled column on the mean ranks with letters (a,b). CR: conventional rice planting system; ORF: organic rice-frog coculture system.

		2013		2014	
		CR	ORF	CR	ORF
		g kg^−1^		g kg^−1^	
P	Soil	**0.073b**	**0.15a**	**0.12b**	**0.21a**
	Straw	1.52a	1.54a	**1.54b**	**2.84a**
	Grain	**2.12b**	**3.14a**	**2.07b**	**3.27a**
K	Soil	0.066a	0.062a	0.045a	0.057a
	Straw	8.67a	11.05a	15.86a	13.75a
	Grain	2.56a	2.93a	2.08a	2.23a
Ca	Soil	**1.79a**	**1.13b**	**1.82a**	**1.12b**
	Straw	5.32a	6.22a	5.08a	4.97a
	Grain	**0.23a**	**0.14b**	**0.37a**	**0.23b**
Mg	Soil	0.21a	0.23a	0.25a	0.31a
	Straw	1.31a	1.73a	2.36a	2.46a
	Grain	0.96a	0.92a	0.95a	1.08a

### Micronutrients concentration in soil and plants

Table 5 shows the concentrations of seven micronutrients in the soil (available concentrations), straw, and grain in the CR and ORF systems. The Cu, B, and Ni concentrations in soil and plant did not differ significantly between the CR and ORF systems. The Mn concentration in soil was 1.30- and 0.87-fold lower in the ORF system than in the CR system in 2013 and 2014, respectively, but the Mn concentration in plants did not differ significantly between the two systems. The Fe concentration in soil and rice straw was significantly higher in the ORF than in the CR system in both years, and the Fe concentration in rice grain was 0.48-fold higher in the ORF system than in the CR system in 2014. The Zn and Mo concentrations in soil and rice grain were significantly higher in the ORF system than in the CR system in both years. In rice straw, the Zn concentration in 2013 and the Mo concentration in 2014 in the ORF system were higher than their correspongding levels in the CR system.

**Table 5:** The effects of CR and ORF cultivation patterns on the micronutrient availabilities in the soil (extracted with 1 M ammonium acetate), concentrations in the shoot and seed at 2013 and 2014. In the table, calculated by ANOVA with Tukey test (p < 0.05), differences between treatments were significantly representing by bold-filled column on the mean ranks with letters (a-b). CR: conventional rice planting system; ORF: organic rice-frog coculture system.

		2013		2014	
		CR	ORF	CR	ORF
		mg kg^−1^		mg kg^−1^	
Mn	**Soil**	**35.59a**	**15.50b**	**35.80a**	**19.13b**
	Straw	489.63a	527.49a	987.91a	1138.29a
	Grain	32.28a	34.00a	47.55a	46.07a
Fe	Soil	**1.02b**	**1.78a**	**1.77b**	**2.94a**
	Straw	**153.27b**	**239.12a**	**299.57b**	**443.36a**
	Grain	9.49a	9.40a	**17.97b**	**26.51a**
Cu	Soil	0.11a	0.11a	0.12a	0.13a
	Straw	2.72a	3.24a	4.64a	4.19a
	Grain	3.57a	3.43a	3.48a	3.79a
Zn	Soil	**0.16b**	**0.30a**	**0.73b**	**1.45a**
	Straw	**24.26b**	**35.52a**	54.50a	57.92a
	Grain	**21.53b**	**27.43a**	**23.22b**	**29.54a**
Mo	Soil	**0.013b**	**0.019a**	**0.007b**	**0.015a**
	Straw	0.80a	1.03a	**0.90b**	**1.88a**
	Grain	**0.26b**	**0.47a**	**0.48b**	**0.89a**
B	Soil	0.15a	0.11a	0.95a	0.92a
	Straw	2.10a	3.05a	8.19a	7.26a
	Grain	0.80a	0.55a	6.60a	6.57a
Ni	Soil	0.056a	0.54a	0.095a	0.11a
	Straw	0.76a	1.36a	4.40a	4.09a
	Grain	0.16a	0.27a	0.40a	0.48a

### Concentrations of nonessential elements in soil and plants

The concentrations of eight nonessential elements in the soil (available concentrations), straw, and grain in the CR and ORF systems are shown in Table 6. Among them, only Sr and As concentrations in soil and plants did not differ significantly between the CR and ORF systems. The Na concentration in soil and rice straw was significantly higher in the ORF system than in the CR system in 2014, but not in 2013. The Se concentration in the soil and rice grain was higher in the ORF system than in the CR system, but that in rice straw was lower in the ORF system than in the CR system. The Se concentration in the rice grain was 3.1- and 1.89-fold higher in the ORF system than in the CR system in 2013 and 2014, respectively. The Al concentration in the soil and grain was significantly lower in the ORF system than in the CR system in both years, and in rice straw in 2013. The Cd concentrations in soil, rice straw, and rice grains were significantly higher in the ORF system than in the CR system. The Cd concentration in rice grains was 4.88- and 4.50-fold higher in the ORF system than in the CR system in 2013 and 2014, respectively. The ORF system significantly increased the Ba concentration in the rice straw and grain in 2013. The Cr concentration in rice grain was 28.41- and 1.07-fold lower in the ORF system than in the CR system in 2013 and 2014, respectively.

**Table 6:** The effects of CR and ORF cultivation patterns on the nonessential elements availabilities in the soil (extracted with 1 M ammonium acetate), concentrations in the shoot and seed at 2013 and 2014.

		2013		2014	
		CR	ORF	CR	ORF
		mg kg^−1^		mg kg^−1^	
Na	Soil	67.44a	72.72a	**63.22b**	**100.65a**
	Straw	14.82a	17.43a	**481.72b**	**561.62a**
	Grain	1.65a	1.47a	26.69a	27.82a
Se	Soil	**0.0015b**	**0.0060a**	**0.0091b**	**0.0297a**
	Straw	**0.10a**	**0.032b**	**0.13a**	**0.086b**
	Grain	**0.010b**	**0.041a**	**0.019b**	**0.055a**
Al	Soil	**1.2a**	**0.17b**	**1.87a**	**0.74b**
	Straw	**152.34a**	**52.35b**	103.19a	86.98a
	Grain	**4.50a**	**1.58b**	**4.62a**	**1.65b**
Sr	Soil	4.14a	4.6a	4.68a	4.96a
	Straw	10.97a	13.69a	16.18a	14.18a
	Grain	0.34a	0.23a	0.46a	0.44a
Cd	Soil	**0.012b**	**0.030a**	**0.006b**	**0.022a**
	Straw	**0.029b**	**0.049a**	**0.11b**	**0.64a**
	Grain	**0.017b**	**0.10a**	**0.02b**	**0.11a**
Ba	Soil	20.04a	24.4.0b	17.58a	15.36a
	Straw	**33.97b**	**47.90a**	46.47a	49.16a
	Grain	**0.37b**	**0.73a**	0.74a	0.74a
Cr	Soil	0.0022a	0.0030a	0.018a	0.013a
	Straw	**6.69b**	**10.80a**	**23.40a**	**19.00b**
	Grain	**0.20a**	**0.0068b**	**0.58a**	**0.28b**
As	Soil	0.013a	0.013a	0.017a	0.015a
	Straw	1.19a	1.34a	2.58a	2.69a
	Grain	0.11a	0.07a	0.19a	0.18a

In the table, calculated by ANOVA with Tukey test (p < 0.05), differences between treatments were significantly representing by bold-filled column on the mean ranks with letters (a-b). CR: conventional rice planting system; ORF: organic rice-frog coculture system.

### Uptake and translocation efficiency to rice grains

**Figure 2:**
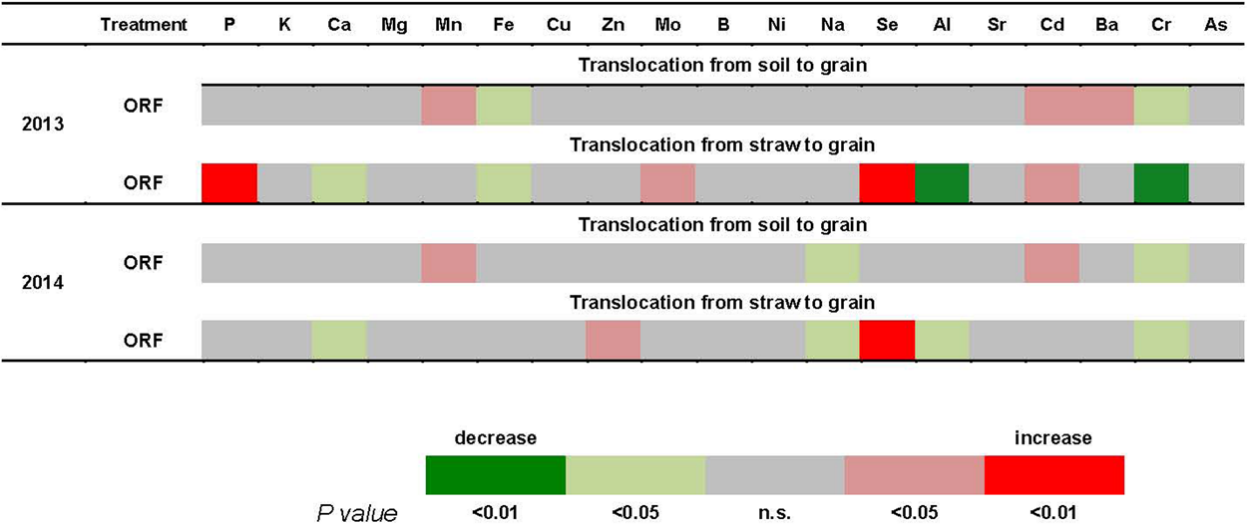
Heat-map showing the relative changes in the translocation ratio of each element from shoot to grain and fro soil to grain in rice grown under ORF compared with CR (CR: conventional rice planting system; ORF: organic rice-frog coculture system). The translocation ratio for each element is the ratio of the concentration in grains to the concentration in the straw and the uptake ratio is the ratio of the concentration in the grains to the available concentration in the soil. For soil, concentrations of 0.2 M ammonium acetate-extractable elements are presented. The red color represents the relative increase and green color the relative decrease. P-value (*P* < 0.05, 0.01) gives the intensity of green and red.

Each element has different availability in soil, and its concentration in the shoot effects its accumulation in grains. Therefore, the effects of the two systems were evaluated for each element as the translocation ratios from soil to grains, and from shoot to grains. Differences in the translocation ratios between ORF and CR are shown as a heat-map in Fig. 2. In 2013, translocation from the soil to grain was significantly increased for Mn, Cd, and Ba, and significantly decreased for Fe and Cr translocation in the ORF system. The translocation from straw to grain was significantly increased for P, Se, Mo, and Cd and decreased for Ca, Fe, Al, and Cr in the ORF system. In 2014, the translocation from soil to grain was increased for Mn and Cd but decreased for Na and Cr in the ORF system. The translocation from straw to grain was increased for Zn and Se but decreased for Ca, Na, Al, and Cr in the ORF system.

### Principal components analysis (PCA) of grain elemental composition

**Figure 3:**
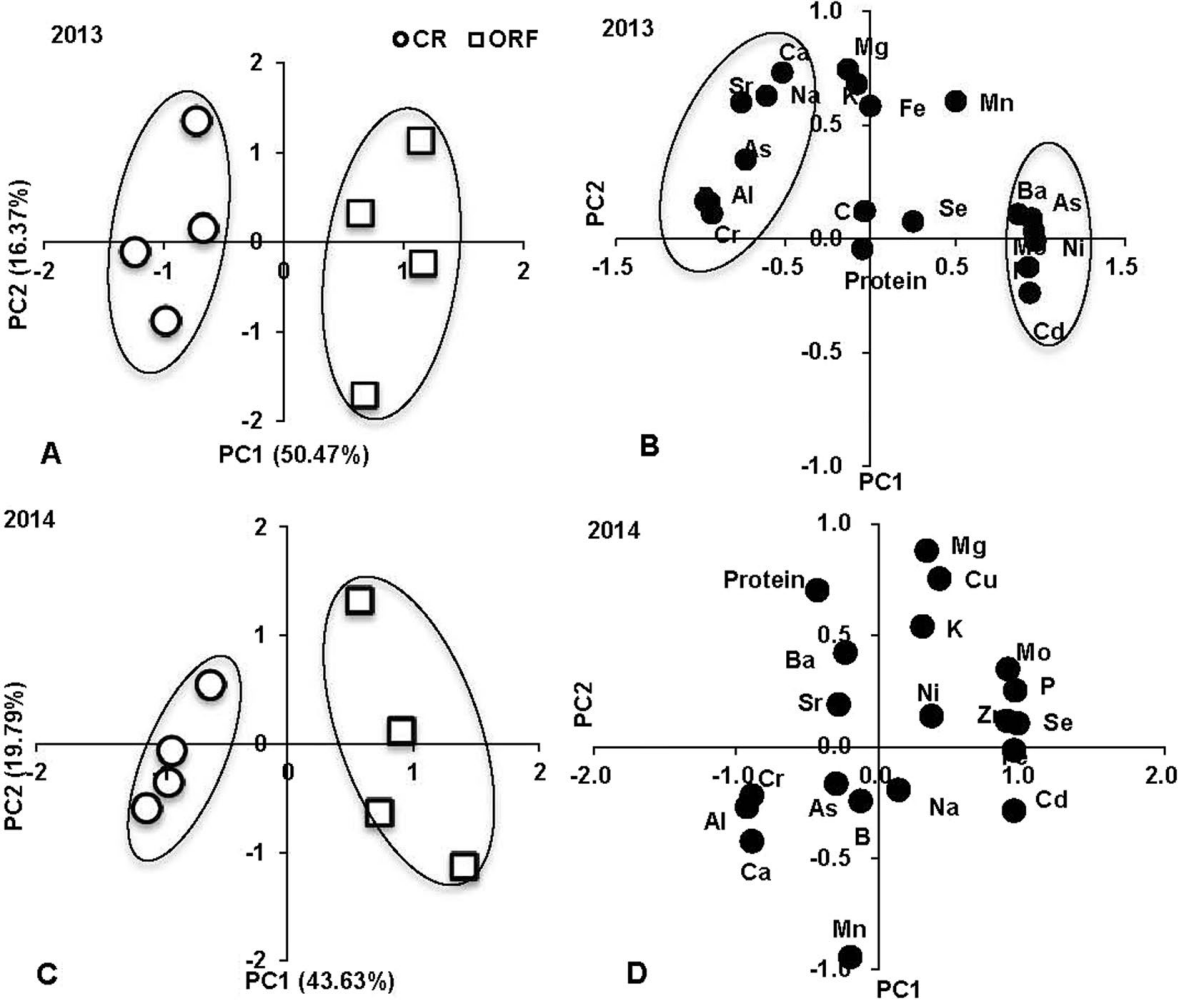
Principle component analysis (PCA) and biplots for protein and ionome concentration in rice grain in response to CR and ORF. (CR: conventional rice planting system; ORF: organic rice-frog coculture system). (**A**) Sample scores for CR and ORF at 2013; (**B**) loading scores of protein and ionome at 2013; (**C**) sample scores for CR and ORF at 2014; (**D**) loading scores of protein and ionome at 2014.

The contents of protein and 19 mineral elements in the rice grain were affected by different cultivation patterns; therefore, these data were subjected to a PCA (Fig. 3). The 20 original variables were converted to a set of two linearly uncorrelated PCs that contributed 66.80% and 63.42% of the total variance in 2013 and 2014, respectively. The difference between CR and ORF was clearly discriminated by PC2 in both years, and PC2 accounted for 16.37% and 19.79% of variation in 2013 and 2014, respectively (Fig. 3A and C). The CR and OFR systems were clearly separated on PC1. The ORF system was located on the positive axis of PC1 with high positive scores; the CR system was located on the negative axis of PC1 with high negative scores. The sample scores of systems reflected the loading score of protein or elements in PCA. In 2013, the high positive sample scores of the ORF system were because of the high positive loading scores: Ba, As, Mo, Ni, P and Cd; and the high negative sample scores of the CR system were due to the high negitive loading scores of Sr, As, B, Al and Cr. Similarly, in 2014, the high positive sample scores of ORF were related to P, Mo, Zn, Fe, Se, and Cd, and the high negative sample scores of CR were related to Cr, Al and Ca.

## Discussion

Frog burrowing in soil can increase the activity of soil enzymes and improve soil aeration, thus increasing the number and growth of soil microbes^32–34^. In our previous study, we reported that the ORF system could effectively control N and P losses from paddy fields, compared with CR system^35^. In another field study conducted at the same time site as our study, introducing frogs into paddy field improved soil nutrient status, and increased the contents of soluble protein, chlorophyll, and soluble sugars in rice leaves, thus increasing rice grain yield^15^. In the present study, we further compared rice yields and elemental composition of rice grain between the ORF and CR systems.

One important result is that the ORF and CR systems had similar grain yields in both years (Fig. 1), confirming that the ORF system, which avoids the excessive use of agro-chemicals, did not reduce yield production. As reviewed by Li *et al*., China has become the world’s leading producer and consumer of pesticides, with production and consumption reaching 265 tons and 179 tons, respectively, in 2011^3^. The large quantities of pesticides applied to agricultural fields and the accumulation of organochlorine and organophosphate pesticides residues have resulted in serious environmental deterioration. Therefore, substituting frogs for pesticides is an important strategy to prevent the accumulation of pesticide residues in soil. Moreover, in the ORF system, the use of organic instead of chemical fertilizer is better for the environment. In China, the overuse of chemical N fertilizers has caused environmental problems due to atmospheric, soil, and water enrichment with reactive N of agricultural origin^4,5,8–10^. The ORF system in this study represhents a feasible strategy to reduce the application of agro-chemicals.

Compared with the CR system, the ORF system significantly increased the P content and reduced the Ca content in rice grain in both years (Tables 4 and 5). The higher P content in the grain was related to the higher available P concentration in soil in the ORF system, and the increased translocation rate of P from straw to grain (Fig. 2). The lower P availability in soil in the CR system might be related to the lower soil pH, because available P is rapidly transformed into immobile forms under low pH^36^. Several studies have shown that the application of organic material can increase P solubility and improve the P status of plants, as compared with chemical fertilizer^37–40^. In the present study, the increase in available P in soil in the ORF system may be because the organic matter added to soil led to a high concentration of organic acids which reduced P sorption in the soil and increased P availability^40,41^. Teng (2016) reported that the ORF system increased the total P content and phosphatase activity in paddy fields^15^. The Ca availability in the paddy soils was reduced in the ORF system, leading to significantly decreased Ca concentration in the rice grain. The decreased Ca availability resulted in lower lower Ca uptake, and a lower translocation rate from straw to grain (Fig. 2), which reduced the Ca concentration in the grain. The lower soil Ca availability might be due to the high concentrations of other cations in the soil. Because Ca is readily replaced by other cations from its binding sites at the exterior surface of the plasma membrane, the Ca requirement increases with increasing concentrations of other cations^42^. In the current study, the ORF system increased the availability of some heavy metals (Tables 5 and 6). The higher soil pH in the ORF system might be another reason for the decreased Ca availability in the soil.

The ORF system significantly affected the availability of some micronutrients. The ORF system decreased Mn availability in soil, but did not affect the Mn accumulation in rice plants. The difference in Mn availability between the CR and ORF systems might be related to soil pH, because Mn is abundant in soils and its availability is mainly controlled by soil pH and redox conditions^42^. Hamnér and Kirchmann (2015) reported that rganic fertilizer application did not significantly affect the Mn concentrations in soil unless the pH varied^28^. The ORF system significantly increased Fe, Zn and Mo accumulation in the grain in one or both years, and increased their concentrations in soil in both years. The lower Fe and Zn concentrations in the grain in the CR system might be because of lower Fe and Zn availability in the soil, resulting from herbicides application. Studies on the effects of herbicides on crop growth have shown that some chemicals, e.g. chlorsulfuron, and diclofop-methyl, can reduce the availability and uptake of Zn and Fe^43,44^. In another study, herbicides had a stronger inhibitory effect on the uptake of nutrients that enter plant roots mainly by diffusion than on the uptake of nutrients that move mainly by mass flow^45^. Thus, using frogs instead of pesticides and herbicides to control weeds and pests has some advantages in terms of the nutritional status of the rice crop.

Adding Zn-rich organic fertilziers may increase the concentration of Zn in crops, because Zn in soil is readily available to plants. The ORF system resulted in higher Mo contents in grain in both years. The ORF system increased Mo availability in soil, and promoted the Mo uptake, and increased its translocation rate from straw to grain (Fig. 2), leading to higher Mo status in grain. The higher soil pH in the ORF system may explain the higher Mo availability in soil. Plants take up Mo in its anion form, and the formation of anion form is negatively correlated with soil pH. Because organic fertilizers gernerally contain very low concentrations of Mo, the effect of organic fertilizers may be negligible unless the soil pH is affected. Hamnér and Kirchmann (2015) reported similar effects of organic amendment on Mo uptake^28^.

Na, Se and Al are generally considered as beneficial elements because they stimulate plant growth, but are not essential. In the current study, the Na contents in soil and straw were significantly enhanced by the ORF system in 2014. This was probably because organic fertilizers were rich in Na, which was readily available to plants in soil (Table 6). The Na concentration in straw and grain was much higher in 2014 than in 2013 in both cultivation systems. The rainfall during the growing season was much higher in 2014 than that in 2013, and this may have increased the availability of some minerals in the soil. The influx of Na^+^ into root is mainly by diffusion, which is affected by the field moisture status. In 2013, the rainfall deficit may have reduced the effect of organic matter on soil Na availability, leading to the similar Na status in plant and soil between CR and ORF systems. The ORF system also increased the available Se concentration in soil, decreased the Se concentration in straw, and increased the Se concentration in grain (Table 6). The decreased Se concentration in the straw was related to the high Se translocation rate from straw to grain (Fig. 2). Although Se is mainly in organic forms in organic fertilizers, it is mineralized to selenite and selenate when organic matter decays, which may explain the increase in Se availability in soil. The effects of organic amendment on crop Se concentrations were paradoxical in previous studies. Some studies reported low bioavailability of Se in organic fertilizers and strong retention of Se by soil organic matter^28,46^. In other studies that used animal manure as a method to increase crop Se content, there were positive correlations between organic matter input and Se uptake in mineral soils^47^. The paradox with respect to the impact of organic matter input on Se uptake may related to competition between S and Se for uptake by plant root, since sulphate is also present in organic fertilizers. We did not measure the S contents in plants and soil in this study, so we can only speculated that mineralized selenite and selenate derived from organic fertilizer competed with S for root uptake. The ORF system significantly decreased the Al concentration in soil and grain in both years (Table 6). The higher Al concentration in soil and grain in the CR system is likely due to the lower soil pH.

Cadmimum, Sr, Ba, Cr, and As are generally considered as toxic elements for plant growth. Although these nonessential metal(loid)s have no known biological functions in plants, they are taken up via essential metal uptake systems^48^. In the current study, the ORF system significantly increased the Ba concentration in the straw and grain in 2013, but did not affect the availability of Ba in soil. The increased Ba uptake by crops might be related to competition between Ca and Ba, since they are chemically similar and share the same translocation pathways^49^. The decrease of Ca uptake in the ORF system (Table 4) suggests that there was same competition for uptake and translocation. A similar competitive relationship exists between Zn and Cd. The ORF system resulted in a significant increase in available Cd content in soil, increased uptake from soil, and increased translocation from straw to grain, leading to high Cd concentrations in the grain in both years (Table 6 and Fig. 2). The opposite patterns were observed for Zn contents in soil and plants. Zinc and Cd compete for ion adsorption and desorption by clay soils, and plant roots can alter the forms of metals (e.g., by oxidation) in the rhizosphere so as to decrease their availability to plants^50^. Because Zn and Cd are chemically similar and readily available to plants in soil, they complete for uptake by the roots – this can explain their opposite patterns of uptake in the ORF system. A previous study also reported paradoxical effects of organic amendments on Cd accumulation.The increase in crop Cd concentrations in fields supplemented with organic matter was probably due to the release of Cd bound to soil organic matter and Fe/Mn-oxides^51^.

The results of the PCA indicated that the ORF system mainly affected Ba, As, Mo, Ni, P, and Cd contents in grain in 2013, and P, Mo, Zn, Fe, Se, and Cd contents in grain in 2014. Thus, the main effect of ORF system on grain was to increase the concentrations of P and trace metal(loid)s. This result was consistent with those of the ANOVA analysis of the ionome of rice grain and soil. The addition of trace elements in organic fertilizer and removal of agro-chemicals were the main reason for variations in the elemental composition of rice grains.

Deficiencies of mineral micronutirents (Fe, Zn, and Se) in the diet affect more than half of the world’s population. More than 60% of the world’s population is Fe deficient, more than 30% is Zn deficient, and 15% is Se deficient^52,53^. Biofortification strategies to increase the concentrations and bioavailability of the elements that are lacking in human diets are a main research focus. Low bioavailability of trace elements in soil is the bottleneck for biofortification^54–60^. The ORF system in this study is one biofortification to increase the micronutrient concentrations in the edible parts of rice.

## Conclusion

The ORF system could maintain rice yield without excessive use of chemical fertilizers, herbicides, or pesticides. Moreover, the ORF system resulted in significant increases in P, Fe, Zn, Mo and Se contents in the rice grain, which may benefit human health. However, the ORF system also increased the concentration of Cd, a potentially toxic elment, in the rice grain, as well as its transport from the soil to the grain. Therefore, ORF system has great potential for sustainable sustainable agriculture development and for preserving resources. However, if this system is adopted for long-term use, more attention should be paid to the risks of higher Cd concentrations in the soil and grain. This is the first study to compare the ionome variations in plants and soil between a rice–frog coculture ecosystem and a conventional farming system.
